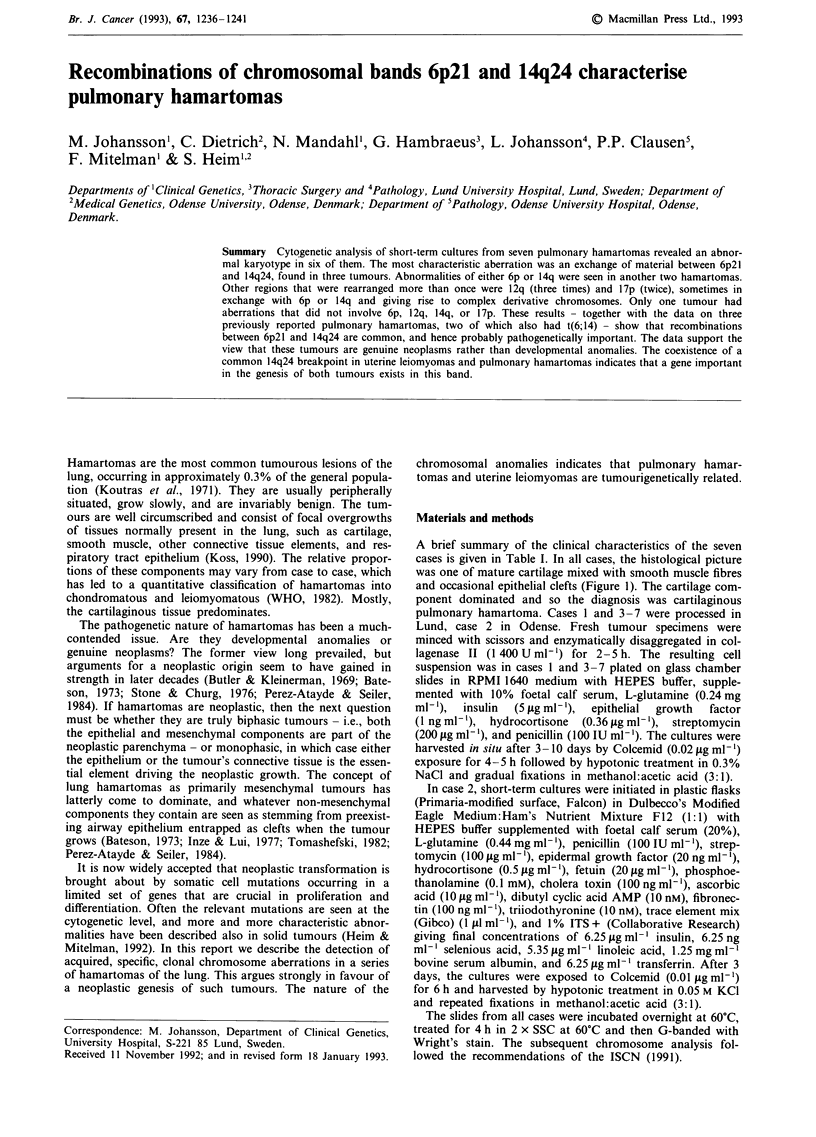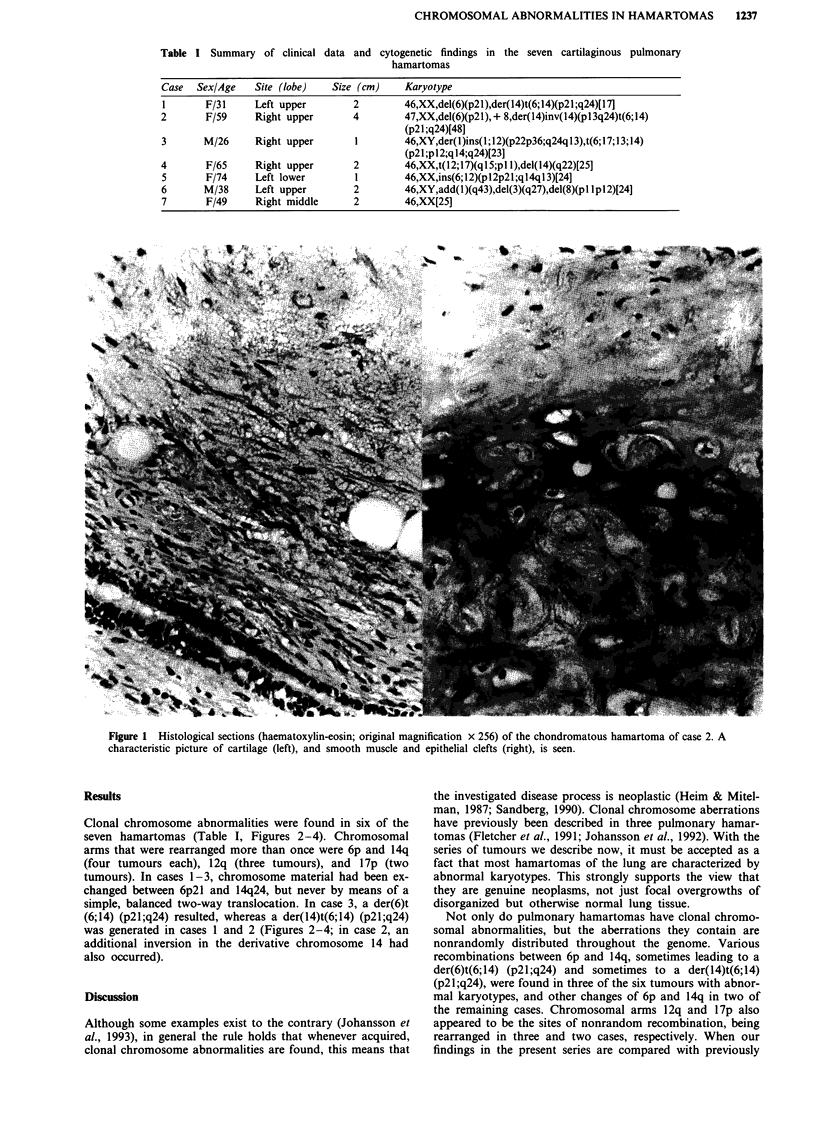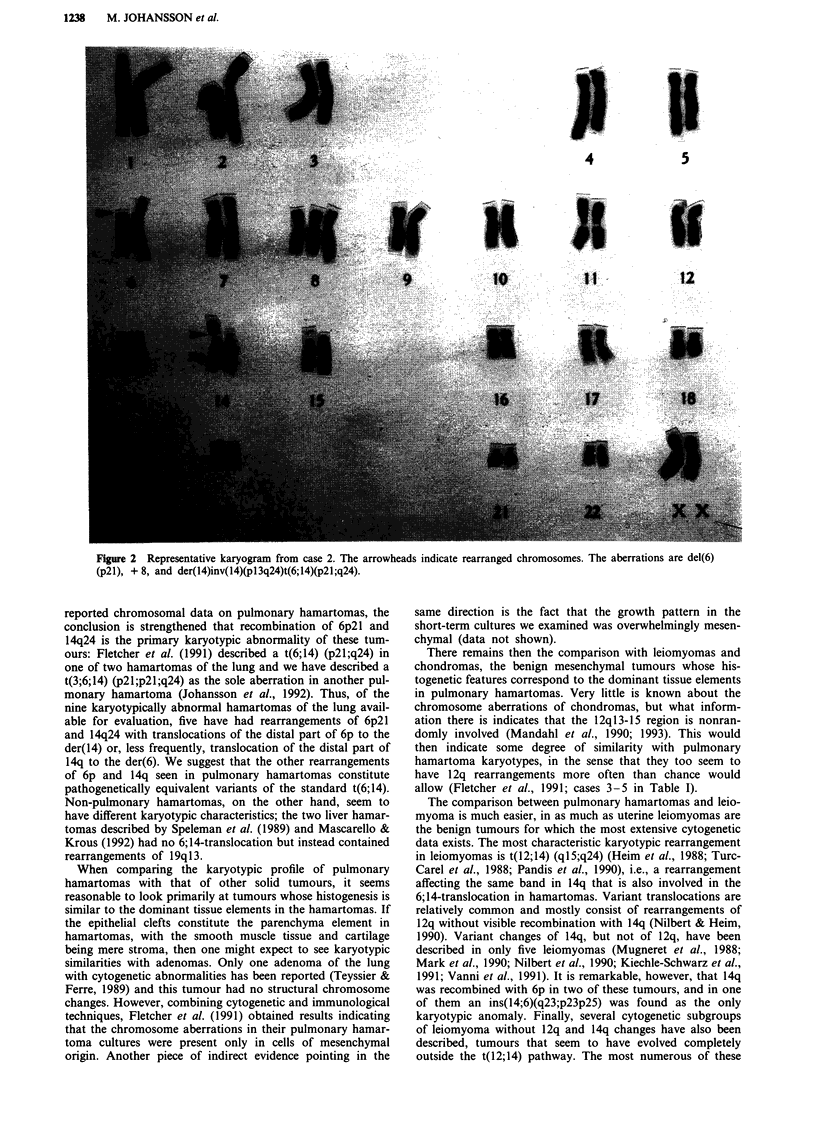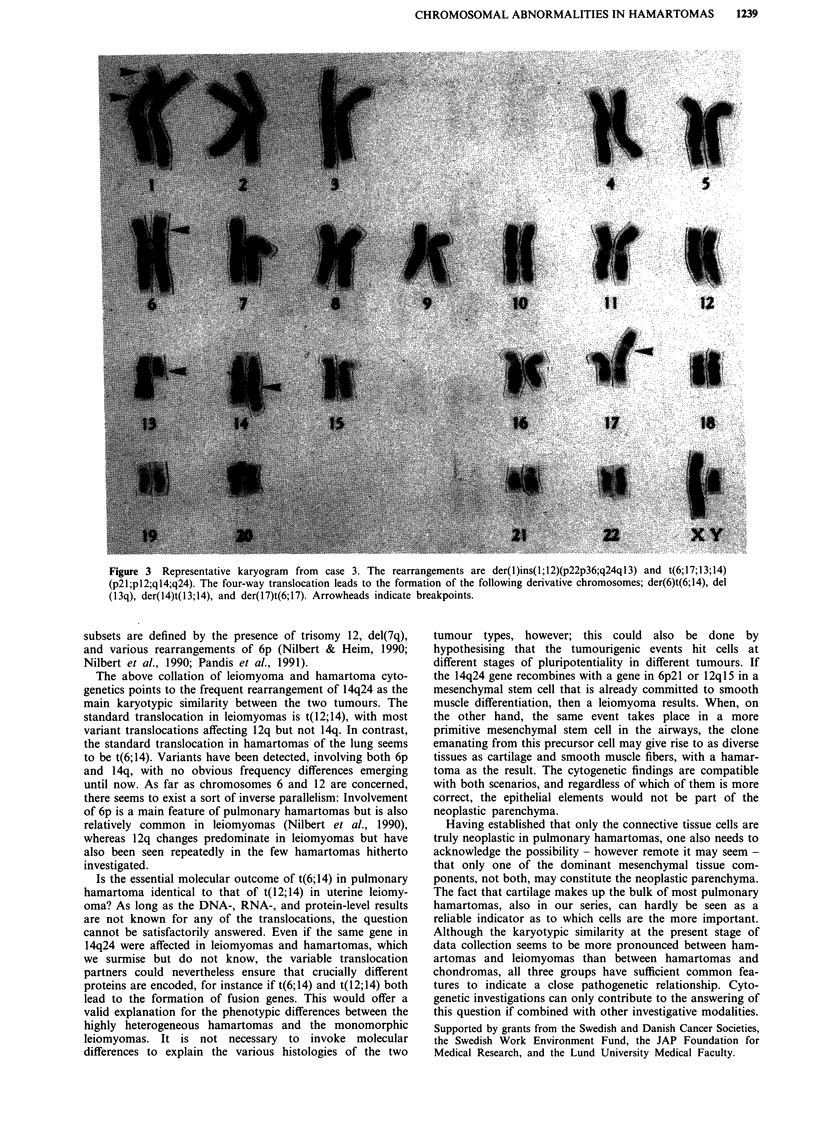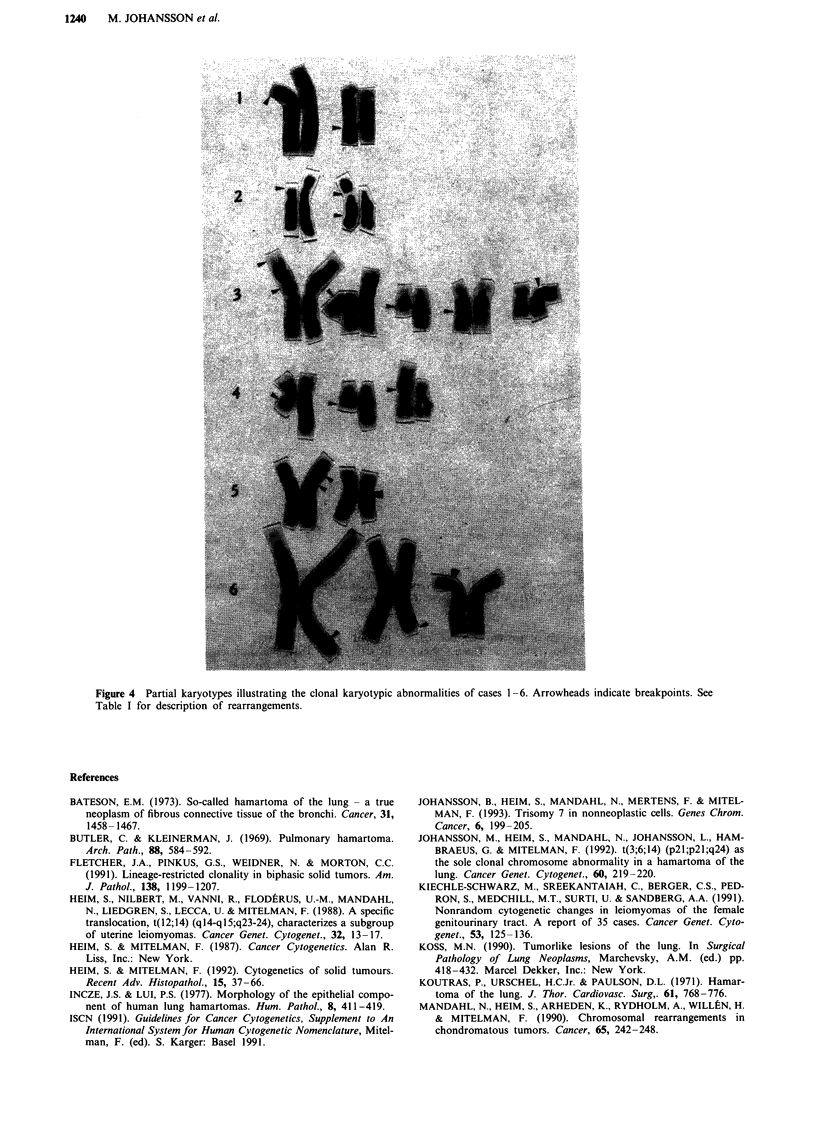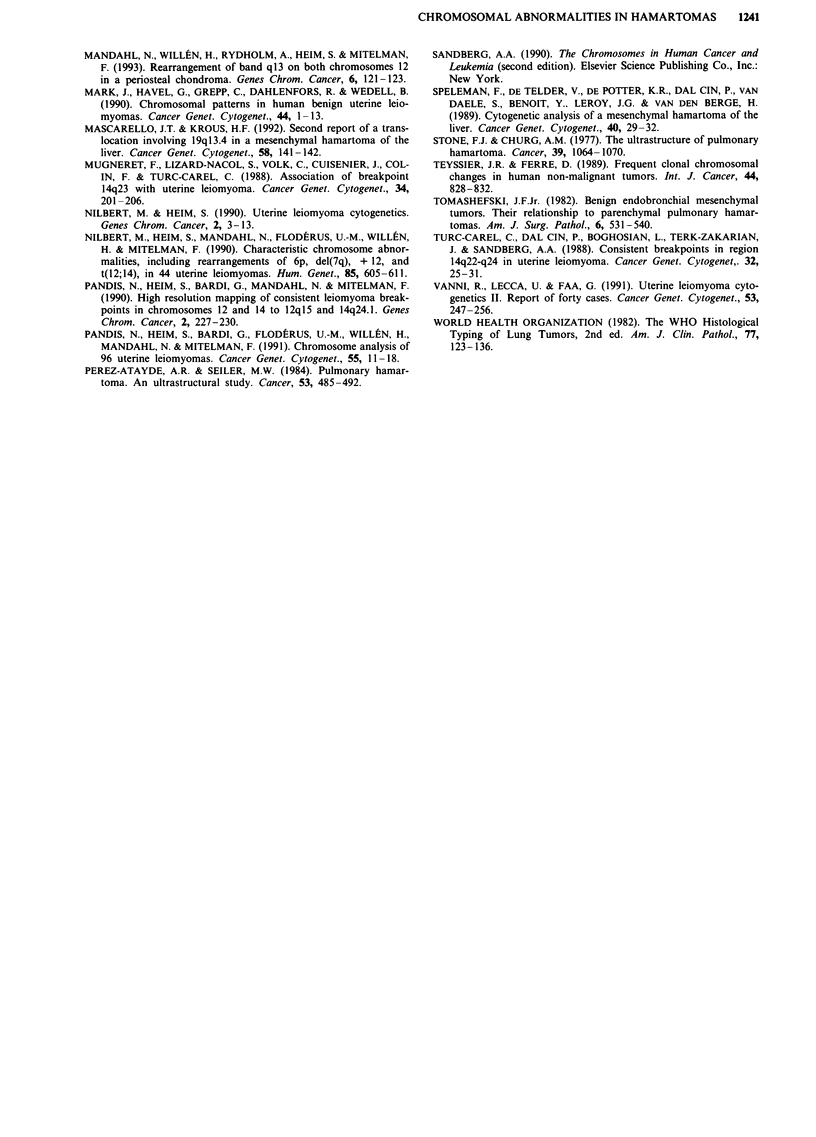# Recombinations of chromosomal bands 6p21 and 14q24 characterise pulmonary hamartomas.

**DOI:** 10.1038/bjc.1993.231

**Published:** 1993-06

**Authors:** M. Johansson, C. Dietrich, N. Mandahl, G. Hambraeus, L. Johansson, P. P. Clausen, F. Mitelman, S. Heim

**Affiliations:** Department of Clinical Genetics, Lund University Hospital, Sweden.

## Abstract

**Images:**


Br. J. Cancer (1993), 67, 1236–1241 © Macmillan Press Ltd., 1993

### Recombinations of chromosomal bands 6p21 and 14q24 characterise pulmonary hamartomas

M. Johansson[1], C. Dietrich[2], N. Mandahl[1], G. Hambraeus[3], L. Johansson[4], P.P. Clausen[5], F. Mitelman[1] & S. Heim[1,2]

*Departments of [1]Clinical Genetics, [3]Thoracic Surgery and [4]Pathology, Lund University Hospital, Lund, Sweden; Department of [2]Medical Genetics, Odense University, Odense, Denmark; Department of [5]Pathology, Odense University Hospital, Odense, Denmark.*

**Summary** Cytogenetic analysis of short-term cultures from seven pulmonary hamartomas revealed an abnormal karyotype in six of them. The most characteristic aberration was an exchange of material between 6p21 and 14q24, found in three tumours. Abnormalities of either 6p or 14q were seen in another two hamartomas. Other regions that were rearranged more than once were 12q (three times) and 17p (twice), sometimes in exchange with 6p or 14q and giving rise to complex derivative chromosomes. Only one tumour had aberrations that did not involve 6p, 12q, 14q, or 17p. These results – together with the data on three previously reported pulmonary hamartomas, two of which also had t(6;14) – show that recombinations between 6p21 and 14q24 are common, and hence probably pathogenetically important. The data support the view that these tumours are genuine neoplasms rather than developmental anomalies. The coexistence of a common 14q24 breakpoint in uterine leiomyomas and pulmonary hamartomas indicates that a gene important in the genesis of both tumours exists in this band.

Hamartomas are the most common tumourous lesions of the lung, occurring in approximately 0.3% of the general population (Koutras *et al.*, 1971). They are usually peripherally situated, grow slowly, and are invariably benign. The tumours are well circumscribed and consist of focal overgrowths of tissues normally present in the lung, such as cartilage, smooth muscle, other connective tissue elements, and respiratory tract epithelium (Koss, 1990). The relative proportions of these components may vary from case to case, which has led to a quantitative classification of hamartomas into chondromatous and leiomyomatous (WHO, 1982). Mostly, the cartilaginous tissue predominates.

The pathogenetic nature of hamartomas has been a much-contended issue. Are they developmental anomalies or genuine neoplasms? The former view long prevailed, but arguments for a neoplastic origin seem to have gained in strength in later decades (Butler & Kleinerman, 1969; Bateson, 1973; Stone & Churg, 1976; Perez-Atayde & Seiler, 1984). If hamartomas are neoplastic, then the next question must be whether they are truly biphasic tumours – i.e., both the epithelial and mesenchymal components are part of the neoplastic parenchyma – or monophasic, in which case either the epithelium or the tumour's connective tissue is the essential element driving the neoplastic growth. The concept of lung hamartomas as primarily mesenchymal tumours has latterly come to dominate, and whatever non-mesenchymal components they contain are seen as stemming from preexisting airway epithelium entrapped as clefts when the tumour grows (Bateson, 1973; Inze & Lui, 1977; Tomashefski, 1982; Perez-Atayde & Seiler, 1984).

It is now widely accepted that neoplastic transformation is brought about by somatic cell mutations occurring in a limited set of genes that are crucial in proliferation and differentiation. Often the relevant mutations are seen at the cytogenetic level, and more and more characteristic abnormalities have been described also in solid tumours (Heim & Mitelman, 1992). In this report we describe the detection of acquired, specific, clonal chromosome aberrations in a series of hamartomas of the lung. This argues strongly in favour of a neoplastic genesis of such tumours. The nature of the chromosomal anomalies indicates that pulmonary hamartomas and uterine leiomyomas are tumourigenetically related.

Correspondence: M. Johansson, Department of Clinical Genetics, University Hospital, S-221 85 Lund, Sweden.
Received 11 November 1992; and in revised form 18 January 1993.

### Materials and methods

A brief summary of the clinical characteristics of the seven cases is given in Table I. In all cases, the histological picture was one of mature cartilage mixed with smooth muscle fibres and occasional epithelial clefts (Figure 1). The cartilage component dominated and so the diagnosis was cartilaginous pulmonary hamartoma. Cases 1 and 3–7 were processed in Lund, case 2 in Odense. Fresh tumour specimens were minced with scissors and enzymatically disaggregated in collagenase II (1 400 U $ml^{-1}$) for 2–5 h. The resulting cell suspension was in cases 1 and 3–7 plated on glass chamber slides in RPMI 1640 medium with HEPES buffer, supplemented with 10% foetal calf serum, L-glutamine (0.24 mg $ml^{-1}$), insulin (5 $\mu g\ ml^{-1}$), epithelial growth factor (1 ng $ml^{-1}$), hydrocortisone (0.36 $\mu g\ ml^{-1}$), streptomycin (200 $\mu g\ ml^{-1}$), and penicillin (100 IU $ml^{-1}$). The cultures were harvested *in situ* after 3–10 days by Colcemid (0.02 $\mu g\ ml^{-1}$) exposure for 4–5 h followed by hypotonic treatment in 0.3% NaCl and gradual fixations in methanol:acetic acid (3:1).

In case 2, short-term cultures were initiated in plastic flasks (Primaria-modified surface, Falcon) in Dulbecco's Modified Eagle Medium:Ham's Nutrient Mixture F12 (1:1) with HEPES buffer supplemented with foetal calf serum (20%), L-glutamine (0.44 mg $ml^{-1}$), penicillin (100 IU $ml^{-1}$), streptomycin (100 $\mu g\ ml^{-1}$), epidermal growth factor (20 ng $ml^{-1}$), hydrocortisone (0.5 $\mu g\ ml^{-1}$), fetuin (20 $\mu g\ ml^{-1}$), phosphoethanolamine (0.1 mM), cholera toxin (100 ng $ml^{-1}$), ascorbic acid (10 $\mu g\ ml^{-1}$), dibutyl cyclic acid AMP (10 nM), fibronectin (100 ng $ml^{-1}$), triiodothyronine (10 nM), trace element mix (Gibco) (1 $\mu l\ ml^{-1}$), and 1% ITS+ (Collaborative Research) giving final concentrations of 6.25 $\mu g\ ml^{-1}$ insulin, 6.25 ng $ml^{-1}$ selenious acid, 5.35 $\mu g\ ml^{-1}$ linoleic acid, 1.25 mg $ml^{-1}$ bovine serum albumin, and 6.25 $\mu g\ ml^{-1}$ transferrin. After 3 days, the cultures were exposed to Colcemid (0.01 $\mu g\ ml^{-1}$) for 6 h and harvested by hypotonic treatment in 0.05 M KCl and repeated fixations in methanol:acetic acid (3:1).

The slides from all cases were incubated overnight at 60°C, treated for 4 h in 2 × SSC at 60°C and then G-banded with Wright's stain. The subsequent chromosome analysis followed the recommendations of the ISCN (1991).

**Table I** Summary of clinical data and cytogenetic findings in the seven cartilaginous pulmonary hamartomas

| *Case* | *Sex/Age* | *Site (lobe)* | *Size (cm)* | *Karyotype* |
|---|---|---|---|---|
| 1 | F/31 | Left upper | 2 | 46,XX,del(6)(p21),der(14)t(6;14)(p21;q24)[17] |
| 2 | F/59 | Right upper | 4 | 47,XX,del(6)(p21), + 8,der(14)inv(14)(p13q24)t(6;14)(p21;q24)[48] |
| 3 | M/26 | Right upper | 1 | 46,XY,der(1)ins(1;12)(p22p36;q24q13),t(6;17;13;14)(p21;p12;q14;q24)[23] |
| 4 | F/65 | Right upper | 2 | 46,XX,t(12;17)(q15;p11),del(14)(q22)[25] |
| 5 | F/74 | Left lower | 1 | 46,XX,ins(6;12)(p12p21;q14q13)[24] |
| 6 | M/38 | Left upper | 2 | 46,XY,add(1)(q43),del(3)(q27),del(8)(p11p12)[24] |
| 7 | F/49 | Right middle | 2 | 46,XX[25] |

**Figure 1** Histological sections (haematoxylin-eosin; original magnification × 256) of the chondromatous hamartoma of case 2. A characteristic picture of cartilage (left), and smooth muscle and epithelial clefts (right), is seen.

### Results

Clonal chromosome abnormalities were found in six of the seven hamartomas (Table I, Figures 2–4). Chromosomal arms that were rearranged more than once were 6p and 14q (four tumours each), 12q (three tumours), and 17p (two tumours). In cases 1–3, chromosome material had been exchanged between 6p21 and 14q24, but never by means of a simple, balanced two-way translocation. In case 3, a der(6)t (6;14) (p21;q24) resulted, whereas a der(14)t(6;14) (p21;q24) was generated in cases 1 and 2 (Figures 2–4; in case 2, an additional inversion in the derivative chromosome 14 had also occurred).

### Discussion

Although some examples exist to the contrary (Johansson *et al.*, 1993), in general the rule holds that whenever acquired, clonal chromosome abnormalities are found, this means that the investigated disease process is neoplastic (Heim & Mitelman, 1987; Sandberg, 1990). Clonal chromosome aberrations have previously been described in three pulmonary hamartomas (Fletcher *et al.*, 1991; Johansson *et al.*, 1992). With the series of tumours we describe now, it must be accepted as a fact that most hamartomas of the lung are characterized by abnormal karyotypes. This strongly supports the view that they are genuine neoplasms, not just focal overgrowths of disorganized but otherwise normal lung tissue.

Not only do pulmonary hamartomas have clonal chromosomal abnormalities, but the aberrations they contain are nonrandomly distributed throughout the genome. Various recombinations between 6p and 14q, sometimes leading to a der(6)t(6;14) (p21;q24) and sometimes to a der(14)t(6;14) (p21;q24), were found in three of the six tumours with abnormal karyotypes, and other changes of 6p and 14q in two of the remaining cases. Chromosomal arms 12q and 17p also appeared to be the sites of nonrandom recombination, being rearranged in three and two cases, respectively. When our findings in the present series are compared with previously

**Figure 2** Representative karyogram from case 2. The arrowheads indicate rearranged chromosomes. The aberrations are del(6)(p21), + 8, and der(14)inv(14)(p13q24)t(6;14)(p21;q24).

reported chromosomal data on pulmonary hamartomas, the conclusion is strengthened that recombination of 6p21 and 14q24 is the primary karyotypic abnormality of these tumours: Fletcher *et al.* (1991) described a t(6;14) (p21;q24) in one of two hamartomas of the lung and we have described a t(3;6;14) (p21;p21;q24) as the sole aberration in another pulmonary hamartoma (Johansson *et al.*, 1992). Thus, of the nine karyotypically abnormal hamartomas of the lung available for evaluation, five have had rearrangements of 6p21 and 14q24 with translocations of the distal part of 6p to the der(14) or, less frequently, translocation of the distal part of 14q to the der(6). We suggest that the other rearrangements of 6p and 14q seen in pulmonary hamartomas constitute pathogenetically equivalent variants of the standard t(6;14). Non-pulmonary hamartomas, on the other hand, seem to have different karyotypic characteristics; the two liver hamartomas described by Speleman *et al.* (1989) and Mascarello & Krous (1992) had no 6;14-translocation but instead contained rearrangements of 19q13.

When comparing the karyotypic profile of pulmonary hamartomas with that of other solid tumours, it seems reasonable to look primarily at tumours whose histogenesis is similar to the dominant tissue elements in the hamartomas. If the epithelial clefts constitute the parenchyma element in hamartomas, with the smooth muscle tissue and cartilage being mere stroma, then one might expect to see karyotypic similarities with adenomas. Only one adenoma of the lung with cytogenetic abnormalities has been reported (Teyssier & Ferre, 1989) and this tumour had no structural chromosome changes. However, combining cytogenetic and immunological techniques, Fletcher *et al.* (1991) obtained results indicating that the chromosome aberrations in their pulmonary hamartoma cultures were present only in cells of mesenchymal origin. Another piece of indirect evidence pointing in the same direction is the fact that the growth pattern in the short-term cultures we examined was overwhelmingly mesenchymal (data not shown).

There remains then the comparison with leiomyomas and chondromas, the benign mesenchymal tumours whose histogenetic features correspond to the dominant tissue elements in pulmonary hamartomas. Very little is known about the chromosome aberrations of chondromas, but what information there is indicates that the 12q13-15 region is nonrandomly involved (Mandahl *et al.*, 1990; 1993). This would then indicate some degree of similarity with pulmonary hamartoma karyotypes, in the sense that they too seem to have 12q rearrangements more often than chance would allow (Fletcher *et al.*, 1991; cases 3–5 in Table I).

The comparison between pulmonary hamartomas and leiomyoma is much easier, in as much as uterine leiomyomas are the benign tumours for which the most extensive cytogenetic data exists. The most characteristic karyotypic rearrangement in leiomyomas is t(12;14) (q15;q24) (Heim *et al.*, 1988; Turc-Carel *et al.*, 1988; Pandis *et al.*, 1990), i.e., a rearrangement affecting the same band in 14q that is also involved in the 6;14-translocation in hamartomas. Variant translocations are relatively common and mostly consist of rearrangements of 12q without visible recombination with 14q (Nilbert & Heim, 1990). Variant changes of 14q, but not of 12q, have been described in only five leiomyomas (Mugneret *et al.*, 1988; Mark *et al.*, 1990; Nilbert *et al.*, 1990; Kiechle-Schwarz *et al.*, 1991; Vanni *et al.*, 1991). It is remarkable, however, that 14q was recombined with 6p in two of these tumours, and in one of them an ins(14;6)(q23;p23p25) was found as the only karyotypic anomaly. Finally, several cytogenetic subgroups of leiomyoma without 12q and 14q changes have also been described, tumours that seem to have evolved completely outside the t(12;14) pathway. The most numerous of these

**Figure 3** Representative karyogram from case 3. The rearrangements are der(1)ins(1;12)(p22p36;q24q13) and t(6;17;13;14)(p21;p12;q14;q24). The four-way translocation leads to the formation of the following derivative chromosomes; der(6)t(6;14), del(13q), der(14)t(13;14), and der(17)t(6;17). Arrowheads indicate breakpoints.

subsets are defined by the presence of trisomy 12, del(7q), and various rearrangements of 6p (Nilbert & Heim, 1990; Nilbert *et al.*, 1990; Pandis *et al.*, 1991).

The above collation of leiomyoma and hamartoma cytogenetics points to the frequent rearrangement of 14q24 as the main karyotypic similarity between the two tumours. The standard translocation in leiomyomas is t(12;14), with most variant translocations affecting 12q but not 14q. In contrast, the standard translocation in hamartomas of the lung seems to be t(6;14). Variants have been detected, involving both 6p and 14q, with no obvious frequency differences emerging until now. As far as chromosomes 6 and 12 are concerned, there seems to exist a sort of inverse parallelism: Involvement of 6p is a main feature of pulmonary hamartomas but is also relatively common in leiomyomas (Nilbert *et al.*, 1990), whereas 12q changes predominate in leiomyomas but have also been seen repeatedly in the few hamartomas hitherto investigated.

Is the essential molecular outcome of t(6;14) in pulmonary hamartoma identical to that of t(12;14) in uterine leiomyoma? As long as the DNA-, RNA-, and protein-level results are not known for any of the translocations, the question cannot be satisfactorily answered. Even if the same gene in 14q24 were affected in leiomyomas and hamartomas, which we surmise but do not know, the variable translocation partners could nevertheless ensure that crucially different proteins are encoded, for instance if t(6;14) and t(12;14) both lead to the formation of fusion genes. This would offer a valid explanation for the phenotypic differences between the highly heterogeneous hamartomas and the monomorphic leiomyomas. It is not necessary to invoke molecular differences to explain the various histologies of the two tumour types, however; this could also be done by hypothesising that the tumourigenic events hit cells at different stages of pluripotentiality in different tumours. If the 14q24 gene recombines with a gene in 6p21 or 12q15 in a mesenchymal stem cell that is already committed to smooth muscle differentiation, then a leiomyoma results. When, on the other hand, the same event takes place in a more primitive mesenchymal stem cell in the airways, the clone emanating from this precursor cell may give rise to as diverse tissues as cartilage and smooth muscle fibers, with a hamartoma as the result. The cytogenetic findings are compatible with both scenarios, and regardless of which of them is more correct, the epithelial elements would not be part of the neoplastic parenchyma.

Having established that only the connective tissue cells are truly neoplastic in pulmonary hamartomas, one also needs to acknowledge the possibility – however remote it may seem – that only one of the dominant mesenchymal tissue components, not both, may constitute the neoplastic parenchyma. The fact that cartilage makes up the bulk of most pulmonary hamartomas, also in our series, can hardly be seen as a reliable indicator as to which cells are the more important. Although the karyotypic similarity at the present stage of data collection seems to be more pronounced between hamartomas and leiomyomas than between hamartomas and chondromas, all three groups have sufficient common features to indicate a close pathogenetic relationship. Cytogenetic investigations can only contribute to the answering of this question if combined with other investigative modalities.

Supported by grants from the Swedish and Danish Cancer Societies, the Swedish Work Environment Fund, the JAP Foundation for Medical Research, and the Lund University Medical Faculty.

**Figure 4** Partial karyotypes illustrating the clonal karyotypic abnormalities of cases 1–6. Arrowheads indicate breakpoints. See Table I for description of rearrangements.

### References

BATESON, E.M. (1973). So-called hamartoma of the lung – a true neoplasm of fibrous connective tissue of the bronchi. *Cancer*, **31**, 1458–1467.

BUTLER, C. & KLEINERMAN, J. (1969). Pulmonary hamartoma. *Arch. Path.*, **88**, 584–592.

FLETCHER, J.A., PINKUS, G.S., WEIDNER, N. & MORTON, C.C. (1991). Lineage-restricted clonality in biphasic solid tumors. *Am. J. Pathol.*, **138**, 1199–1207.

HEIM, S., NILBERT, M., VANNI, R., FLODÉRUS, U.-M., MANDAHL, N., LIEDGREN, S., LECCA, U. & MITELMAN, F. (1988). A specific translocation, t(12;14) (q14-q15;q23-24), characterizes a subgroup of uterine leiomyomas. *Cancer Genet. Cytogenet.*, **32**, 13–17.

HEIM, S. & MITELMAN, F. (1987). *Cancer Cytogenetics*. Alan R. Liss, Inc.: New York.

HEIM, S. & MITELMAN, F. (1992). Cytogenetics of solid tumours. *Recent Adv. Histopathol.*, **15**, 37–66.

INCZE, J.S. & LUI, P.S. (1977). Morphology of the epithelial component of human lung hamartomas. *Hum. Pathol.*, **8**, 411–419.

ISCN (1991). *Guidelines for Cancer Cytogenetics, Supplement to An International System for Human Cytogenetic Nomenclature*, Mitelman, F. (ed). S. Karger: Basel 1991.

JOHANSSON, B., HEIM, S., MANDAHL, N., MERTENS, F. & MITELMAN, F. (1993). Trisomy 7 in nonneoplastic cells. *Genes Chrom. Cancer*, **6**, 199–205.

JOHANSSON, M., HEIM, S., MANDAHL, N., JOHANSSON, L., HAMBRAEUS, G. & MITELMAN, F. (1992). t(3;6;14) (p21;p21;q24) as the sole clonal chromosome abnormality in a hamartoma of the lung. *Cancer Genet. Cytogenet.*, **60**, 219–220.

KIECHLE-SCHWARZ, M., SREEKANTAIAH, C., BERGER, C.S., PEDRON, S., MEDCHILL, M.T., SURTI, U. & SANDBERG, A.A. (1991). Nonrandom cytogenetic changes in leiomyomas of the female genitourinary tract. A report of 35 cases. *Cancer Genet. Cytogenet.*, **53**, 125–136.

KOSS, M.N. (1990). Tumorlike lesions of the lung. In *Surgical Pathology of Lung Neoplasms*, Marchevsky, A.M. (ed.) pp. 418–432. Marcel Dekker, Inc.: New York.

KOUTRAS, P., URSCHEL, H.C.Jr. & PAULSON, D.L. (1971). Hamartoma of the lung. *J. Thor. Cardiovasc. Surg*,. **61**, 768–776.

MANDAHL, N., HEIM, S., ARHEDEN, K., RYDHOLM, A., WILLÉN, H. & MITELMAN, F. (1990). Chromosomal rearrangements in chondromatous tumors. *Cancer*, **65**, 242–248.

MANDAHL, N., WILLÉN, H., RYDHOLM, A., HEIM, S. & MITELMAN, F. (1993). Rearrangement of band q13 on both chromosomes 12 in a periosteal chondroma. *Genes Chrom. Cancer*, **6**, 121–123.

MARK, J., HAVEL, G., GREPP, C., DAHLENFORS, R. & WEDELL, B. (1990). Chromosomal patterns in human benign uterine leiomyomas. *Cancer Genet. Cytogenet.*, **44**, 1–13.

MASCARELLO, J.T. & KROUS, H.F. (1992). Second report of a translocation involving 19q13.4 in a mesenchymal hamartoma of the liver. *Cancer Genet. Cytogenet.*, **58**, 141–142.

MUGNERET, F., LIZARD-NACOL, S., VOLK, C., CUISENIER, J., COLIN, F. & TURC-CAREL, C. (1988). Association of breakpoint 14q23 with uterine leiomyoma. *Cancer Genet. Cytogenet.*, **34**, 201–206.

NILBERT, M. & HEIM, S. (1990). Uterine leiomyoma cytogenetics. *Genes Chrom. Cancer*, **2**, 3–13.

NILBERT, M., HEIM, S., MANDAHL, N., FLODÉRUS, U.-M., WILLÉN, H. & MITELMAN, F. (1990). Characteristic chromosome abnormalities, including rearrangements of 6p, del(7q), + 12, and t(12;14), in 44 uterine leiomyomas. *Hum. Genet.*, **85**, 605–611.

PANDIS, N., HEIM, S., BARDI, G., MANDAHL, N. & MITELMAN, F. (1990). High resolution mapping of consistent leiomyoma breakpoints in chromosomes 12 and 14 to 12q15 and 14q24.1. *Genes Chrom. Cancer*, **2**, 227–230.

PANDIS, N., HEIM, S., BARDI, G., FLODÉRUS, U.-M., WILLÉN, H., MANDAHL, N. & MITELMAN, F. (1991). Chromosome analysis of 96 uterine leiomyomas. *Cancer Genet. Cytogenet.*, **55**, 11–18.

PEREZ-ATAYDE, A.R. & SEILER, M.W. (1984). Pulmonary hamartoma. An ultrastructural study. *Cancer*, **53**, 485–492.

SANDBERG, A.A. (1990). *The Chromosomes in Human Cancer and Leukemia* (second edition). Elsevier Science Publishing Co., Inc.: New York.

SPELEMAN, F., DE TELDER, V., DE POTTER, K.R., DAL CIN, P., VAN DAELE, S., BENOIT, Y., LEROY, J.G. & VAN DEN BERGE, H. (1989). Cytogenetic analysis of a mesenchymal hamartoma of the liver. *Cancer Genet. Cytogenet.*, **40**, 29–32.

STONE, F.J. & CHURG, A.M. (1977). The ultrastructure of pulmonary hamartoma. *Cancer*, **39**, 1064–1070.

TEYSSIER, J.R. & FERRE, D. (1989). Frequent clonal chromosomal changes in human non-malignant tumors. *Int. J. Cancer*, **44**, 828–832.

TOMASHEFSKI, J.F.Jr. (1982). Benign endobronchial mesenchymal tumors. Their relationship to parenchymal pulmonary hamartomas. *Am. J. Surg. Pathol.*, **6**, 531–540.

TURC-CAREL, C., DAL CIN, P., BOGHOSIAN, L., TERK-ZAKARIAN, J. & SANDBERG, A.A. (1988). Consistent breakpoints in region 14q22-q24 in uterine leiomyoma. *Cancer Genet. Cytogenet*,. **32**, 25–31.

VANNI, R., LECCA, U. & FAA, G. (1991). Uterine leiomyoma cytogenetics II. Report of forty cases. *Cancer Genet. Cytogenet.*, **53**, 247–256.

WORLD HEALTH ORGANIZATION (1982). The WHO Histological Typing of Lung Tumors, 2nd ed. *Am. J. Clin. Pathol.*, **77**, 123–136.